# Prospective learning curve analysis of en bloc resection of bladder tumor using an ex vivo porcine model

**DOI:** 10.1186/s12893-024-02355-w

**Published:** 2024-02-19

**Authors:** Qiu Yao, Huizhong Jiang, Hui Niu, Guangmo Hu, Xiaolong Liu, Boxin Xue

**Affiliations:** 1 Department of Urology, The Second Affiliated Hospital of Soochow University, No. 1055 Sanxiang Road, Suzhou, 215000 Jiangsu China; 2 Department of Operating Room, The Second Affiliated Hospital of Soochow University, Jiangsu, China; 3 Department of Pathology, The Second Affiliated Hospital of Soochow University, Jiangsu, China

**Keywords:** Ex vivo model, Non–muscle-invasive bladder cancer, *En bloc* resection of bladder tumor, Learning curve, Cumulative sum analysis

## Abstract

**Background:**

As a relatively new surgical technique, the learning curve of *en bloc* resection of bladder tumor (ERBT) in ex vivo models remains unaddressed. This study aimed to explore the learning curve of ERBT in an ex vivo porcine model.

**Methods:**

In this prospective study, eight endoscopists without prior experience in ERBT were divided into two groups: junior endoscopists, with less than 100 transurethral resection of bladder tumor (TURBT) procedure experience, and senior endoscopists, with at least 100 TURBT procedure experience. Each endoscopist performed 30 ERBT procedures on artificial lesions in an ex vivo porcine bladder model. The procedure time, perforation, *en bloc* resection status, and absence of detrusor muscle (DM) were recorded. The inflection points were identified using cumulative sum (CUSUM) analysis. Procedure results were compared between the two phases and two groups.

**Results:**

In all, 240 artificial lesions were successfully resected using ERBT. The CUSUM regression line indicated the inflection point at the 16th procedure for the junior endoscopists and at the 13th procedure for the senior endoscopists. In both groups, the procedure time, perforation, piecemeal resection, and DM absence rates were significantly lower in the consolidation phase than in the initial phase. The procedure time for the senior endoscopists was lower than for the junior endoscopists in both phases.

**Conclusions:**

ERBT performance improved significantly after reaching the inflection point of the learning curve in the ex vivo model. We recommend a minimum of 16 ERBT procedures in ex vivo models for urologists with less than 100 TURBT experience and a minimum of 13 procedures for those with at least 100 TURBT experience before advancing to live animal training or supervised clinical practice.

## Background

Recently, there has been an increased interest in *en bloc* resection of bladder tumor (ERBT) because of its potential benefits for treating non-muscle-invasive bladder cancer compared to conventional transurethral resection of bladder tumor (TURBT). First, ERBT involves *en bloc* resection of the bladder tumor along with its base and surrounding tissues, adhering to oncological surgery principles and potentially reducing the tumor reimplantation risk. Additionally, it provides a complete tumor specimen with higher detrusor muscle (DM) presence rates, allowing precise histopathological assessments [1]. Moreover, ERBT can be performed with other energy sources (i.e., Ho:YAG laser, Tm:YAG laser, and hybrid knife) besides conventional electrocautery, potentially reducing obturator nerve reflex and bleeding complications [2–4].

Despite the potential benefits of ERBT for non-muscle-invasive bladder cancer treatment, it is a relatively new surgical technique requiring practice and training to ensure safe and adequate tumor resection. Several training models and simulations have been adopted for conventional TURBT [5–8], but the learning curve (LC) for ERBT in an ex vivo model has yet to be explored. Therefore, this study aimed to evaluate the LC for ERBT using an ex vivo porcine model.

## Methods

**Fig. 1:**
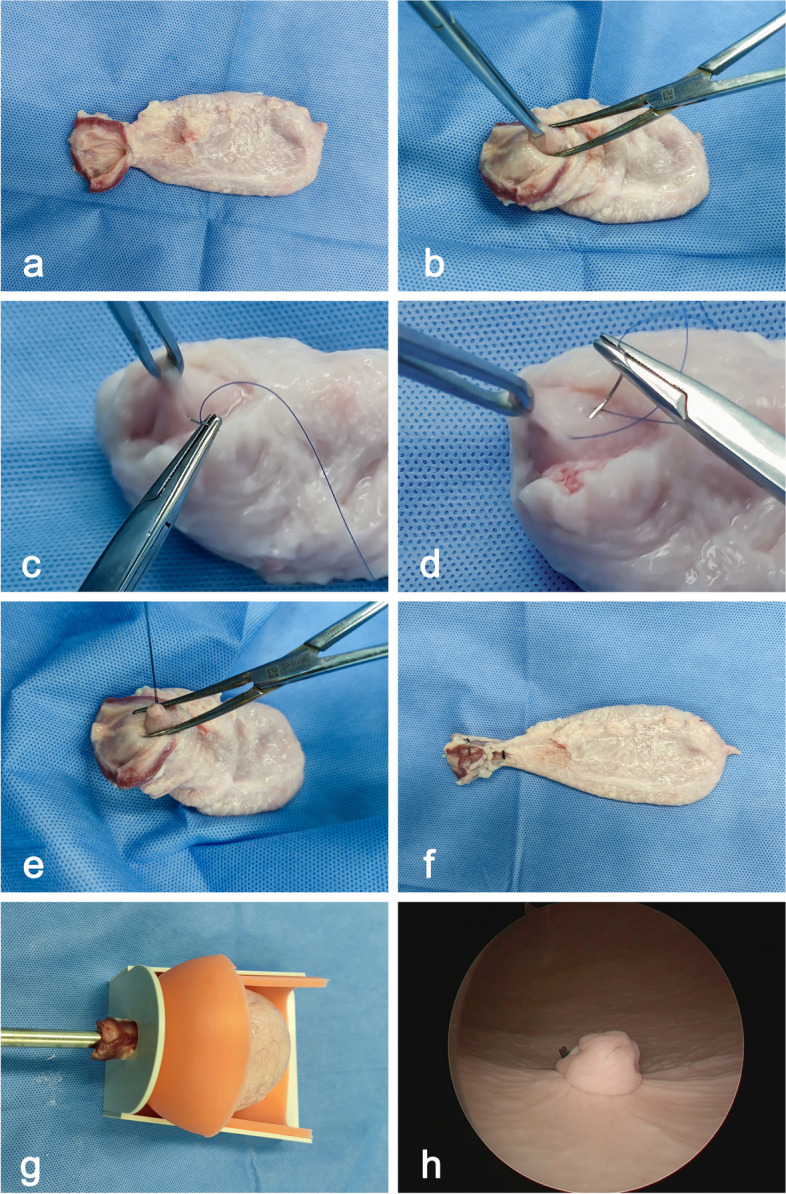
Preparation of the ex vivo porcine training model. **a** An anterior longitudinal incision of the urethra was made from the orifice to the bladder neck region. **b** The mucosa was lifted with forceps. **c** Sutures were applied around the raised mucosa, ensuring an appropriate depth within the submucosal layer. **d** Several submucosal sutures were arranged in a circular pattern to outline the artificial lesion. **e** The sutures were tied off, completing the establishment of the artificial lesion. **f** The incision was closed with sutures. **g** The bladder was placed in the training box, and a resectoscope was inserted through the urethra into the bladder. **h** Endoscopic view of the artificial lesion

This prospective study was conducted at the Urological Endoscopic Training Center of the Second Affiliated Hospital of Soochow University over a period of 6 months. The ex vivo porcine bladders were obtained from a local abattoir. A training model was prepared specifically for this study (Fig. 1). The study involved eight endoscopists who performed 240 ERBT procedures (30 each). None of the endoscopists had prior experience with ERBT, but four of them had experience performing at least 100 TURBT procedures (senior endoscopists), while the remaining four had experience with less than 100 TURBT procedures (junior endoscopists). Participants underwent a tutorial session with an ERBT expert before the study commenced.

### Ex vivo porcine training model

An ex vivo porcine bladder was utilized to create the training model. A longitudinal incision was made in the urethra from the orifice to the bladder neck region (Fig. 1a). The bladder mucosa was lifted through the incision using forceps (Fig. 1b). Sutures were applied around the raised mucosa, ensuring an appropriate depth within the submucosal layer (Fig. 1c). Several submucosal sutures were arranged in a circular pattern to outline the artificial lesion (Fig. 1d). The sutures were tied off, completing the establishment of the artificial lesion (Fig. 1e). The incision was closed with sutures (Fig. 1f). To ensure comparability between procedures, we standardized the size and location of the lesions by creating a single lesion with a diameter of approximately 1.0 cm in either the trigone (Fig. 1h) or the lateral wall of the bladder.

A pelvic simulation training box with an entry on the bottom was prepared. The box comprised two layers: a hard outer wall, for easy placement on the operating table, and a soft layer beneath for stabilizing the specimens inside. The ex vivo bladder was placed in the training box, with the urethra threaded through the entry site. This allowed the insertion of a resectoscope into the bladder via the urethra (Fig. 1g).

### ERBT protocols

**Fig. 2:**
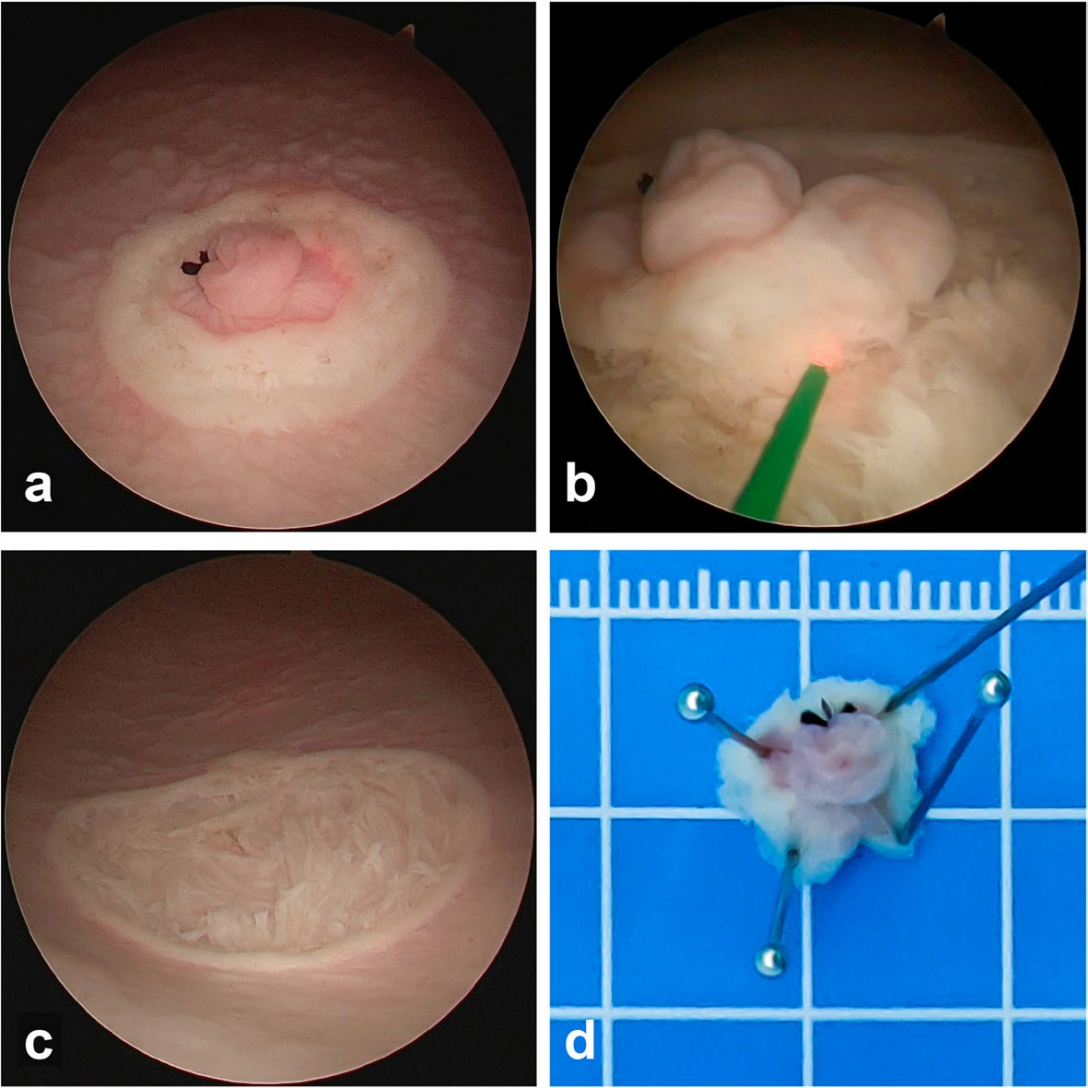
*En bloc* resection of bladder tumor procedure protocols. **a** Circumferential incision around the bladder lesion. **b** The lesion was dissected between the muscular fibers. **c** Change in the bladder wall after lesion removal. **d** Specimen pinned on a plate

All surgeries were performed using the standard ERBT protocols [9]. A continuous-flow laser resectoscope (Olympus Winter *&* Ibe Gmbh, Hamburg, Germany) and a holmium laser system (VersaPulse PowerSuite, Lumenis, Yokneam, Israel) were employed for all procedures. The laser energy settings ranged from 1.0 to 2.0 Joules, with a frequency range of 20 to 40 Hz. These settings were adjustable, allowing the surgeons to tailor them according to their preferences and procedural requirements. During the ERBT procedures, normal saline was used as the irrigation fluid and positioned at a height of 45–70 cm above the operating table. The height was adjusted accordingly to ensure a clear view and maintain suitable bladder capacity during the operation. First, a circumferential incision was made with the holmium laser on the bladder mucosa around the “tumor” at a distance of 5–10 mm (Fig. 2a). Second, the incision was deepened into the DM layer, making the muscle fibers visible. The lesion and surrounding tissue were then dissected between the muscular fibers using the laser (Fig. 2b). The specimen was removed through the resectoscope sheath. Finally, the resection bed was checked using endoscopy. The change in the bladder wall after lesion removal is shown in Fig. 2c.

### Data collection

**Fig. 3:**
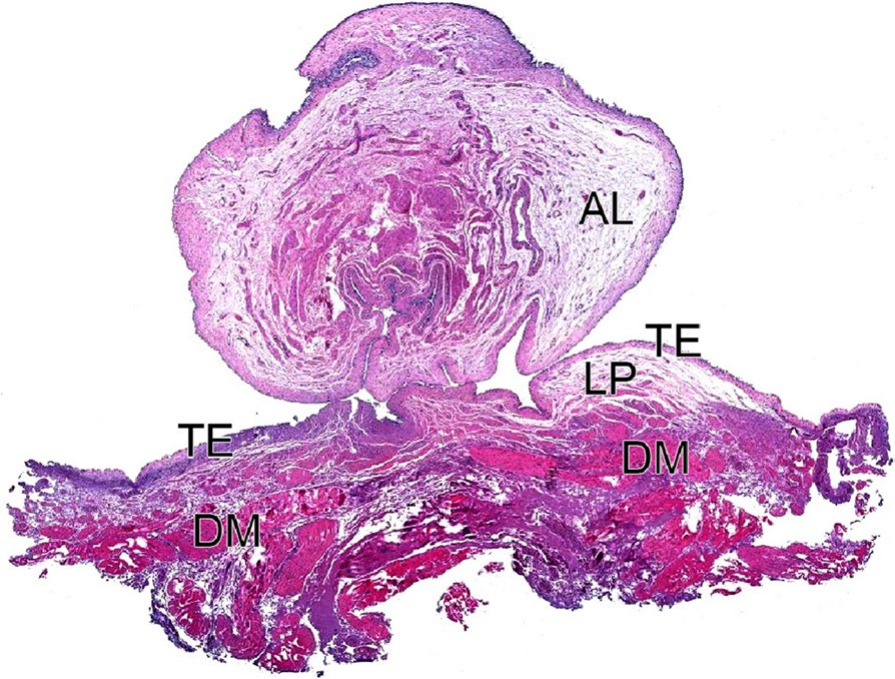
Photomicrograph of a specimen that was endoscopically resected using *en bloc* resection of bladder tumor. Identification of bladder layers: AL, artificial lesions; TE, transitional epithelium; LP, lamina propria; DM, detrusor muscle

Data on procedure time, perforation, and *en bloc* resection status were recorded by an independent observer. After resection, the specimens were arranged on a flat plastic plate, secured with pins (Fig. 2d), immersed in formalin solution, and sent to a pathologist to confirm the presence of DM (Fig. 3). Procedure time was measured from the start of the circumferential incision to the removal of the artificial lesions. To assess perforation, an endoscopic view of a hole in the bladder wall or water leakage through the bladder wall during or after the procedure was considered evidence of perforation.

### Statistical analysis

We selected a sample size of 30 procedures based on the available literature on simulator training for TURBT and endoscopic submucosal dissection [10, 11]. Continuous variables are presented as mean ± standard deviation and were analyzed using Student’s t-test. Categorical variables are presented as frequencies and percentages and were compared using either the chi-squared test or Fisher’s exact test. Statistical significance was set at *p* < 0.05. Statistical analyses were conducted using the SPSS software (version 26.0; SPSS, IBM Company, Armonk, NY, USA).

To evaluate the LC, we used the procedure time as a measure of the efficiency of the serial procedures. Procedure times were graphed in aggregates against serial procedures using polynomial regression. The inflection point of the curve was identified to determine the transition from the learning to the consolidation phase. The line before the inflection point usually had a negative slope, indicating the initial learning phase, whereas the section after the turning point was generally flat, indicating the consolidation phase.

To determine the inflection point in the LC, the cumulative sum (CUSUM) method was used, a widely used technique for evaluating surgical training [12, 13]. CUSUM was calculated as the total running time of the differences between individual procedure times and the mean procedure time for all procedures. For the first procedure, CUSUM was calculated as the difference between the procedure time and the mean procedure time. Subsequent CUSUMs were calculated by adding the CUSUM from the previous procedure to the difference for the current procedure. This process was repeated until the CUSUM for the final procedure equaled zero. The resulting CUSUM scores were plotted against the sequential procedures to identify the inflection point, defined as the point at which the curve started to gradually decline, indicating the required number of procedures to master ERBT in the ex vivo model.

## Results

All 240 artificial lesions were successfully resected using the ERBT technique. Each endoscopist performed 30 ERBT procedures, and the mean procedure time was 35.85 ± 15.47 min. Perforations occurred in 26 procedures (10.83%); these were repaired with sutures, and the ERBT technique was used to complete the procedures. Sixteen lesions (6.67%) were not fully resected *en bloc* and were removed in pieces. DM sampling was achieved in 228 procedures (95.00%).

**Fig. 4:**
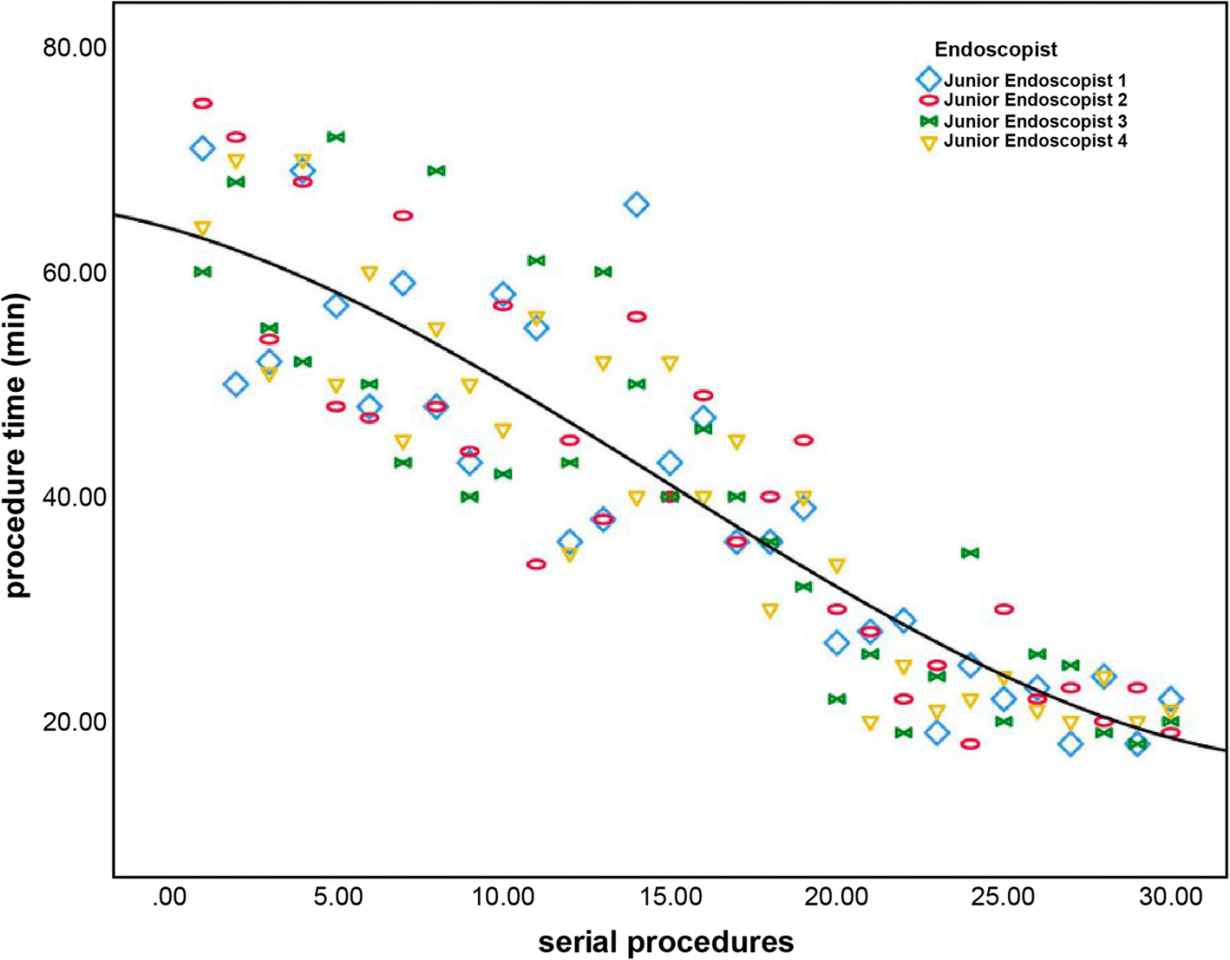
Aggregated procedure time (junior endoscopists) presented via serial procedures. The aggregated line displays a reduction in average procedure time

**Fig. 5:**
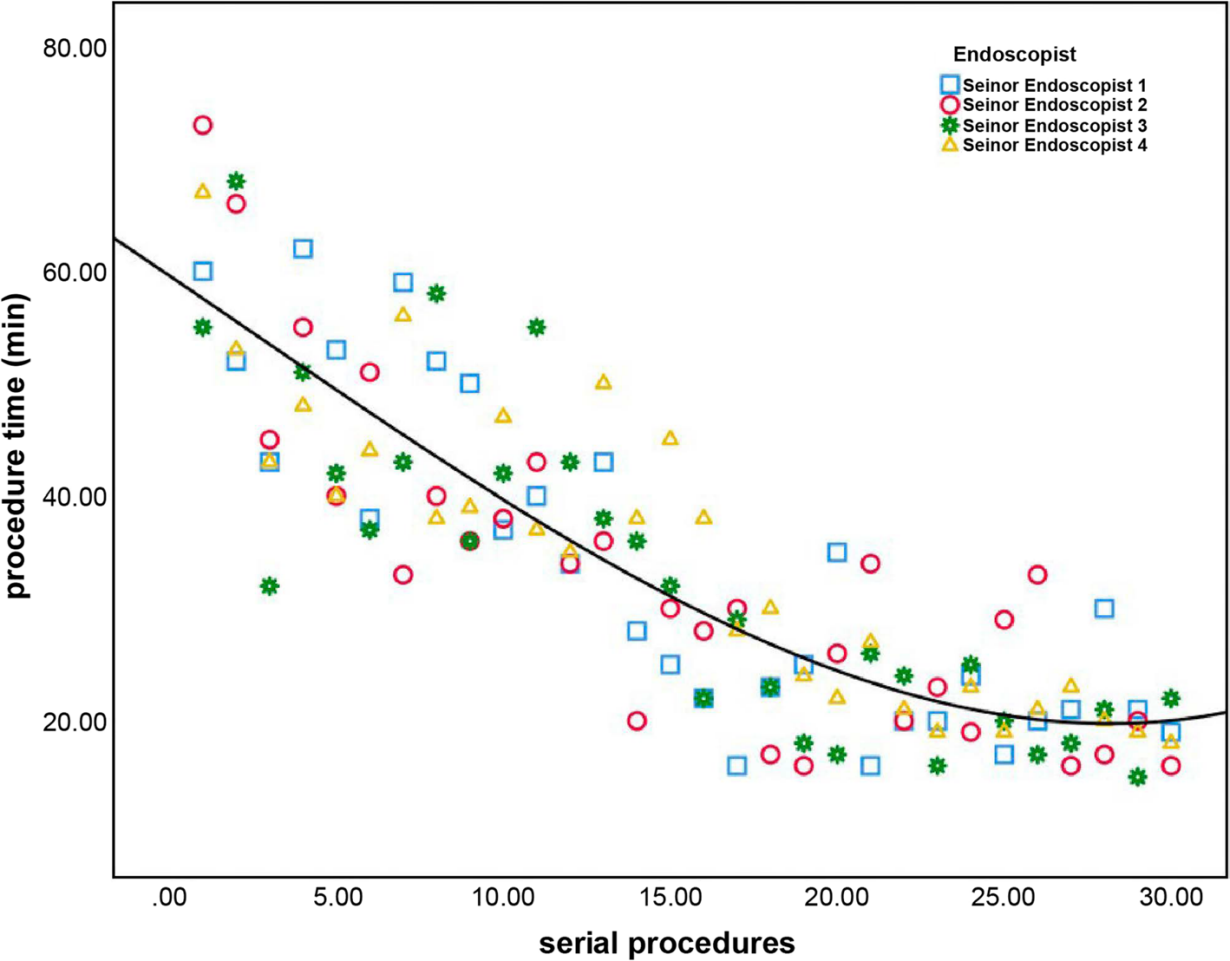
Aggregated procedure time (senior endoscopists) presented via serial procedures. The aggregated line displays a reduction in average procedure time

**Fig. 6:**
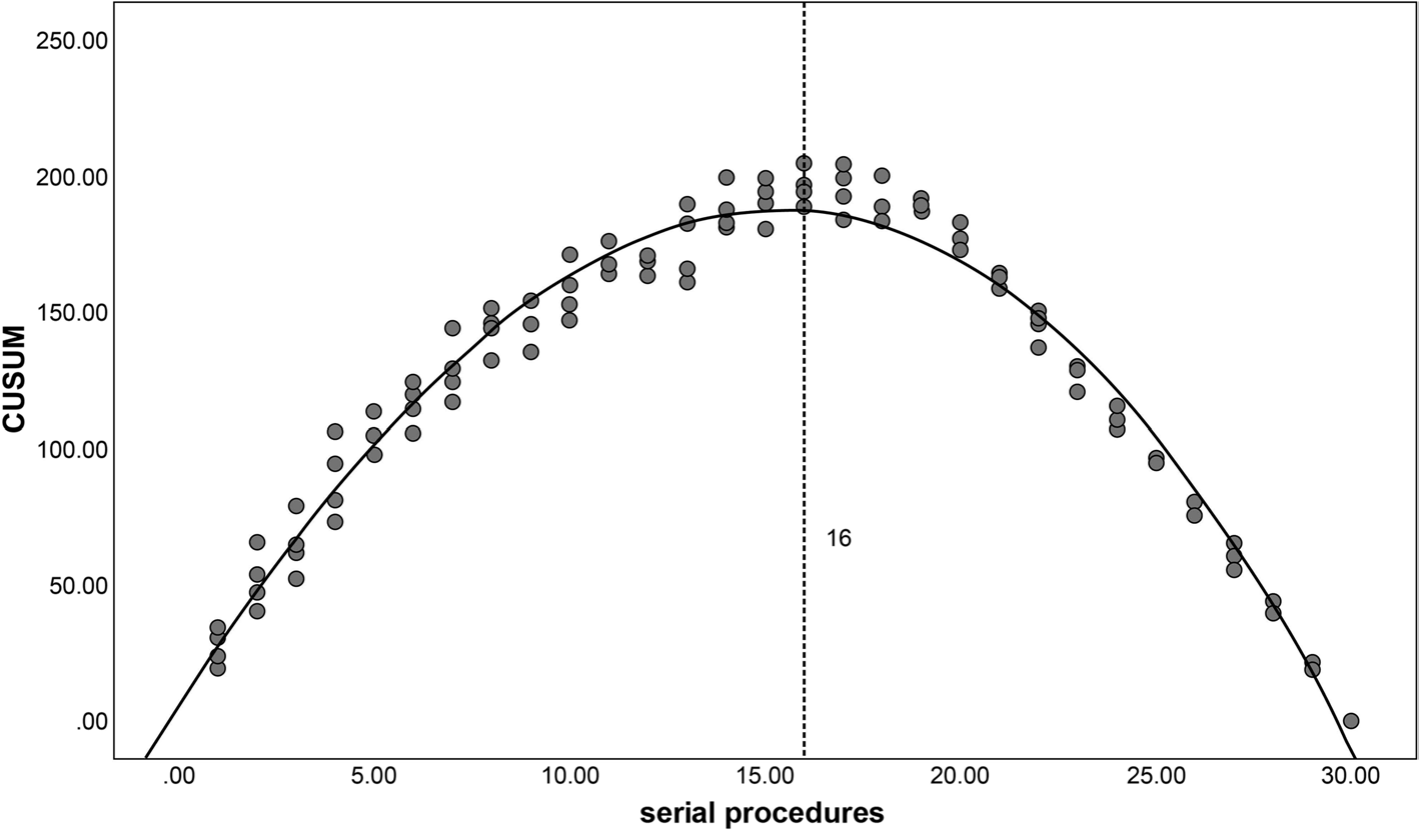
Aggregated cumulative sum (junior endoscopists) presented via serial procedures. The inflection point is denoted by the dashed line

**Fig. 7:**
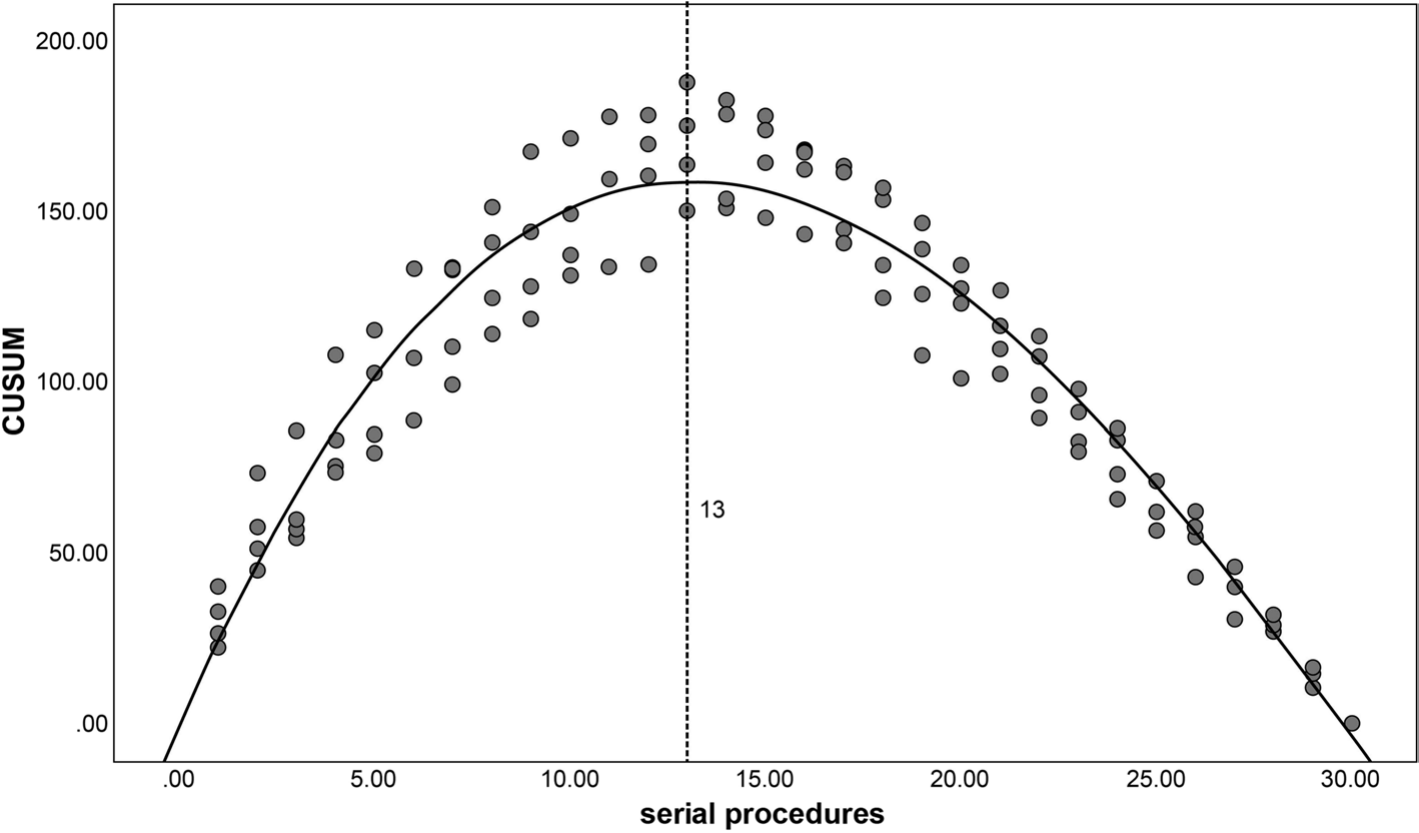
Aggregated cumulative sum (senior endoscopists) presented via serial procedures. The inflection point is denoted by the dashed line

The endoscopists observed downward trends in procedure time over the course of the study. The aggregate data for procedure times for the junior endoscopists and the senior endoscopists is summarized in Figs. 4 and 5. The CUSUM regression line showed that the inflection point of the LC for the junior endoscopists was the 16th procedure (Fig. 6), while for the senior endoscopists the inflection point was the 13th procedure (Fig. 7). The cut-off point divided the LC into two phases. For the junior endoscopists, the initial phase was the first 16 procedures, and the consolidation phase was the following 14 procedures. As for the senior endoscopists, the initial phase was the first 13 procedures, and the consolidation phase was the following 17 procedures.

As shown in Table 1, the procedure time, perforation rate, piecemeal resection rate, and DM absence rate were significantly lower in the consolidation phase than in the initial phase (*p* < 0.05) for both groups. In Table 2, the senior endoscopist group had significantly lower procedure time than the junior endoscopist group in both phases (*p* < 0.05). However, there were no significant differences in the perforation rate, piecemeal resection rate, and DM absence rate between the two groups in both phases.

**Table 1:** *En bloc* resection of bladder tumor procedure parameters for two learning phases

Variables	Junior endoscopists				Senior endoscopists	
	Initial phase (*n* = 64)	Consolidation phase (*n* = 56)	*p*-value	Initial phase (*n* = 52)	Consolidation phase (*n* = 68)	*p*-value
Procedure time (min)	52.61 ± 10.67	26.36 ± 7.39	0.000	46.35 ± 10.17	23.41 ± 6.37	0.000
Perforation: % (n)	25.00% (16/64)	1.79% (1/56)	0.000	17.31% (9/52)	0 (0/68)	0.000
Piecemeal resection: % (n)	15.63% (10/64)	0 (0/56)	0.002	11.54% (6/52)	0 (0/68)	0.004
DM absent: % (n)	9.38% (6/64)	0 (0/56)	0.019	11.54% (6/52)	0 (0/68)	0.004

*DM* Detrusor muscle

**Table 2:** *En bloc* resection of bladder tumor procedure results for two groups

Variables	Initial phase			Consolidation phase		
	Junior endoscopists (*n* = 64)	Senior endoscopists (*n* = 52)	*p*-value	Junior endoscopists (*n* = 56)	Senior endoscopists (*n* = 68)	*p*-value
Procedure time (min)	52.61 ± 10.67	46.35 ± 10.17	0.002	26.36 ± 7.39	23.41 ± 6.37	0.019
Perforation: % (n)	25.00% (16/64)	17.31% (9/52)	0.316	1.79% (1/56)	0 (0/68)	0.269
Piecemeal resection: % (n)	15.63% (10/64)	11.54% (6/52)	0.526	0 (0/56)	0 (0/68)	—
DM absent: % (n)	9.38% (6/64)	11.54% (6/52)	0.704	0 (0/56)	0 (0/68)	—

*DM* Detrusor muscle

## Discussion

In terms of pathological staging, and surgical and oncologic outcomes, ERBT offers potential advantages over conventional TURBT [14–16]. However, its technical complexity and LC may hinder its adoption in clinical practice. Enhancing training opportunities are essential for broader applications.

Acquiring ERBT skills involves an LC, during which endoscopists’ dissection speed, accuracy, and safety gradually improve. For safety considerations, initial training for this technique should be performed using models or simulators. The use of ex vivo porcine specimens for ERBT training is important because it provides haptic properties similar to those of human tissue during lesion dissection, unlike synthetic material models.

It has been reported that various simulation training programs have positively impacted TURBT training [5–8]. However, the high cost of the simulators has limited their widespread implementation. Teoh et al. [17] developed a cost-effective and simple ex vivo porcine bladder training model for TURBT and suggested its suitability for ERBT training. However, there is currently no published research on the LC of ERBT using an ex vivo model.

There are several limitations to using an ex vivo porcine model for ERBT training. First, the model does not simulate bleeding or obturator reflex, which are important complications encountered by endoscopists during ERBT. Second, compared to human bladders, porcine bladders are thinner and lack peri-vesical fat, which can make achieving en bloc resection without perforation more challenging. However, these differences can also be advantageous for training. Third, the artificial lesions used in this study could not fully simulate natural bladder tumors as actual bladder tumors differ in size and location. ERBT can be performed for lesions smaller than 3 cm in size, and the entire specimen can be excised in one piece [18, 19]. Tumors arising from the anterior and posterior bladder walls or the dome are more challenging to resect [20, 21]. Therefore, standardized lesion training only helps trainees acquire basic ERBT skills. Additional training using models with different lesion sizes and locations as well as mentored case-based training are still necessary for endoscopists to become fully competent in this technique.

We used procedure time as the primary measure of surgical skill to assess the LC, instead of using the Objective Structured Assessment for Transurethral Resection of Bladder Tumors Skills (OSATURBS) tool or a combined score [8, 10], for several reasons. First, TURBT is a common procedure routinely performed by urologists [22], and our study primarily focused on learning *en bloc* resection skills rather than other skills that are equally required for TURBT. Second, procedure time directly reflects surgical proficiency, which is a key marker of competence and has been widely used as a reliable metric for predicting LC [23–26]. Third, the procedure time can be easily and accurately recorded and requires no complex data transformation to plot the LC. However, safety and quality of the resection are also important factors in the assessment of skill acquisition. To evaluate quality, we used the *en bloc* resection and DM absence rates as proxies, while the perforation rate was used to evaluate safety.

Throughout our study, we observed significant improvements in ERBT performance, with the inflection point of the LC occurring after the completion of 16 ERBT procedures for the junior endoscopists and 13 procedures for the senior endoscopists. This inflection point holds clinical significance, as it is associated with a substantial reduction in procedure time and notable enhancements in surgical outcomes. Notably, we observed a significant decrease in the perforation rate, indicating a lower risk of complications. Moreover, there were improvements in the rates of *en bloc* resection and DM sampling, indicating enhanced surgical precision and quality.

Although ERBT is a relatively new procedure, it shares certain steps and principles with TURBT. Given that it has been established that a minimum of 100 cases of TURBT is required for a resident in training to achieve satisfactory surgical and oncological outcomes [27], we categorized senior endoscopists separately from junior endoscopists at the threshold of 100 cases. Our study demonstrated that prior experience in TURBT positively influenced the number of procedures required to reach the inflection point of LC and the procedure time during ERBT performance. However, the difference between junior and senior endoscopists in the initial phase appears modest. This observation can be attributed to several factors: Firstly, despite variations in their experience with TURBT, all endoscopists were novices in ERBT. Secondly, senior endoscopists had prior experience with TURBT utilizing electrocautery. However, in our study, we employed laser for ERBT, which introduced a relatively new technique, instrument, and energy source to even the experienced endoscopists. 

While learning a new surgical technique, it is difficult to avoid mistakes. Unfortunately, these errors can compromise the quality and safety of the procedure, making it unreasonable and unethical to practice directly on human patients. Using an ex vivo training model allows surgeons to safely make mistakes and learn, thereby reducing the risk of errors during actual procedures. Although live animal training can help surgeons prepare for intraprocedural complications such as bleeding, perforation, or obturator reflex, it is significantly more expensive and presents ethical complexities compared to the use of ex vivo specimens.

## Conclusions

Our study demonstrated the LC for performing ERBT using an ex vivo model among endoscopists with different experience in TURBT and confirmed the validity of the model. Based on our findings, we recommend that urologists with less than 100 TURBT experience perform a minimum of 16 procedures and urologists with at least 100 TURBT experience perform a minimum of 13 procedures using ex vivo models before advancing to live animal training or supervised clinical performance.

## Data Availability

The data analyzed in this study are available from the corresponding author upon reasonable request.

## Abbreviations

CUSUM: Cumulative sum
DM: Detrusor muscle
ERBT: *en bloc* Resection of bladder tumor
LC: Learning curve
TURBT: Transurethral resections of bladder tumor
